# Green and Efficient Extraction of *Taraxacum kok-saghyz* Natural Rubber and Its Structural Analysis

**DOI:** 10.3390/ijms26030920

**Published:** 2025-01-22

**Authors:** Jiagang Zheng, Fuquan Zhang, Qingyun Zhao, Rentong Yu, Yanfang Zhao, Xiaoxue Liao, Lusheng Liao

**Affiliations:** 1 School of Materials Science and Engineering, Hainan University, Haikou 570228, China; 15103664831@163.com (J.Z.); 18216994049@163.com (Q.Z.); rentong.yu@hainu.edu.cn (R.Y.); zhaoyanfang818@163.com (Y.Z.); 2 Agricultural Products Processing Research Institute of Chinese Academy of Tropical Agricultural Sciences, Hainan Key Laboratory of Natural Rubber Processing, Zhanjiang 524001, China; xqcy12106@163.com

**Keywords:** *Taraxacum kok-saghyz*, natural rubber, extraction, isolation, structure

## Abstract

Natural rubber (NR) is in high demand due to its excellent elasticity and physical and mechanical properties, but production is limited and NR is in short supply. There is an urgent need to find new alternative rubber sources. *Taraxacum kok-saghyz* (TKS), as a green, renewable, widely planted and high content rubber producing plant, has shown broad application prospects. The extraction process is the key to developing efficient, green, and high-purity *Taraxacum kok-saghyz* Natural Rubber (TKNR) to replace NR in various applications. In this study, TKS roots were processed through repeated boiling to remove inulin, followed by alkaline treatment with potassium hydroxide (KOH) to isolate lignin and facilitate cell wall disruption. Subsequent enzymatic hydrolysis using pectinase and cellulase enabled the dissolution of root-structure carbohydrates, thereby obtained TKNR. Structural characterization of TKNR was conducted and compared with that of NR. The results showed that the combined alkaline and enzymatic extraction methodology effectively isolates TKNR from TKS roots. Structural analysis reveals that TKNR closely resembles NR, having comparable molecular weight and distribution, crystallinity, and crosslinking networks, with both polymers primarily consisting of cis-1,4-polyisoprene.

## 1. Introduction

Natural rubber (NR), primarily consisting of *cis*-1,4-polyisoprene, is a naturally synthesized polymer predominantly derived from the *Hevea brasiliensis* tree. However, rising demand for NR, coupled with challenges such as price volatility, restricted cultivation regions, susceptibility to environmental stressors, pest infestations, and lengthy maturation periods, has heightened the need for alternative NR sources [1]. *Taraxacum kok-saghyz* (TKS), a green and high-yielding rubber-producing plant, presents an appealing alternative due to its ease of cultivation, rapid harvest cycles, and broad adaptability to various geographic regions [2,3]. The main rubber component in TKS is similar to the molecular structure and physical–mechanical properties of Brazilian Natural Rubber (NR), highlighting the potential of TKS as an alternative source of NR [4,5,6].

*Taraxacum kok-saghyz* Natural Rubber (TKNR) is primarily found in the roots of TKS in latex form, mirroring the structure of NR [7,8,9]. The TKNR content in TKS roots ranges from 2.8% to 28.7%, influenced by factors such as plant strain, cultivation environment, and diurnal temperature variation [10,11,12,13]. David A. Ramirez-Cadavid and colleagues have conducted extensive research on TKNR extraction methods, initially developing a solvent-based extraction technique to isolate rubber from *Taraxacum kok-saghyz* roots (TK) [14]. Subsequently, they advanced a novel aqueous extraction method, and more recently, an alkaline pretreatment approach for TKNR extraction [15,16]. Additionally, Shomaila Sikandar et al. identified the thermophilic fungus *STm* as a source of hydrolytic enzymes that facilitate TKNR extraction from TKS using enzymatic hydrolysis [17]. Shuai Zhao and co-researchers developed an extraction method that leverages yeast fermentation to simultaneously produce TKNR and biofuel ethanol from TKS [18]. Collectively, these advancements have accelerated the progress toward industrial-scale TKNR extraction.

However, several challenges remain. For example, while solvent extraction is effective, it is constrained by high costs, environmental risks, and safety concerns. Aqueous extraction, though promising, can disrupt TKNR’s network structure, requires substantial water resources, and yields TKNR of lower purity. Alkaline and enzymatic extraction methods, while less disruptive, still result in relatively low purity and efficiency [19], leaving room for optimization in terms of yield and cost-effectiveness [20,21]. In TKNR extraction, yield and efficiency are essential parameters, as the composition and quality of the extract directly impact TKNR performance, serving as primary metrics for assessing the effectiveness of the extraction process [22,23,24].

This study evaluates TKNR extraction using two approaches: an alkaline extraction method and a combined approach integrating alkaline treatment with enzymatic hydrolysis. Structural analysis of the extracted TKNR was performed using Fourier-transform infrared spectroscopy (FTIR), nuclear magnetic resonance (NMR), crosslink density measurement, and X-ray photoelectron spectroscopy (XPS). Thermal stability, glass transition temperature, and crystallinity were further assessed through thermogravimetric analysis (TGA) and differential scanning calorimetry (DSC). The results demonstrate that TKNR extracted using both methods exhibits molecular structures and crosslinking networks highly similar to those of NR. However, TKNR extracted through alkali pretreatment combined with enzymatic hydrolysis displays a more intact structure, closely resembling that of NR. The green and efficient extraction method combining alkaline treatment and enzymatic hydrolysis offers significant advantages, providing a theoretical foundation for the industrial production of TKNR.

## 2. Results and Discussion

### 2.1. Analysis of Rubber Molecular Structure

**Figure 1:**
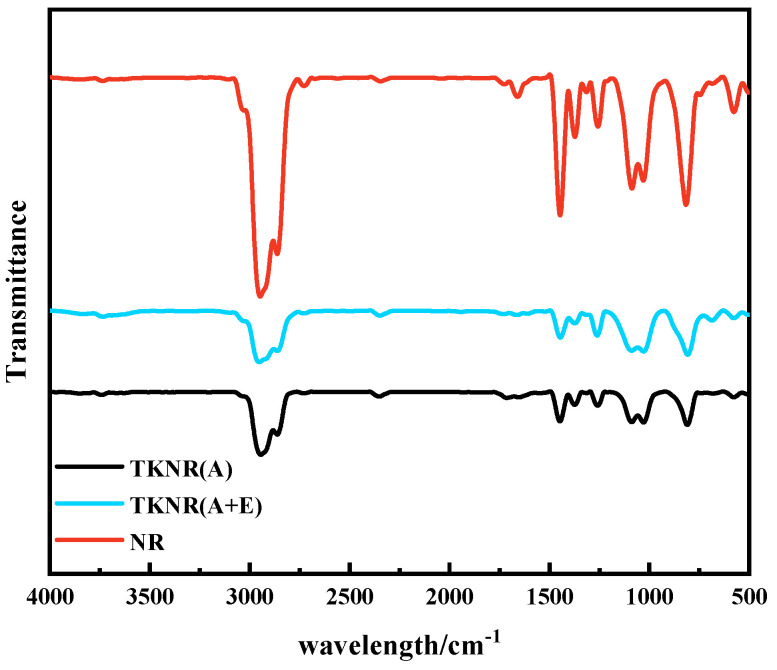
FTIR spectra of NR and TKNR. (A + E) refers to the alkali–enzyme method for extracting TKNR, while (A) refers to the alkali method for extracting TKNR.

Figure 1 presents the FTIR spectra of NR and TKNR, with characteristic absorption peaks of TKNR observed at 1376 cm^−1^, corresponding to the symmetric deformation vibration of methyl groups, and at 836 cm^−1^, indicating the out-of-plane deformation of the C–H bond in cis-disubstituted carbon–carbon double bonds. These peaks are also characteristic of NR and are commonly used to identify the structural features of polyisoprene. Specifically, the peak at 1376 cm^−1^ reflects the methyl group vibrations typical of polyisoprene, while the peak at 836 cm^−1^ is indicative of the cis-1,4 configuration, which distinguishes natural rubber and its derivatives from synthetic forms. Additional peaks at 2962 cm^−1^, 2928 cm^−1^, and 1449 cm^−1^correspond to the asymmetric stretching vibration of CH_3_, the asymmetric stretching vibration of CH_2_, and the antisymmetric deformation vibration of methylene, respectively. The close alignment of these peaks with those in NR indicates that TKNR possesses a structure identical to NR, characterized by a high content of rubber hydrocarbons. However, certain peaks in the TKNR spectrum, including those at 1376 cm^−1^ and 836 cm^−1^, exhibit reduced intensities relative to NR, likely due to partial molecular chain degradation or a reduction in functional groups during alkaline or alkaline–enzymatic extraction processes [25].

**Figure 2:**
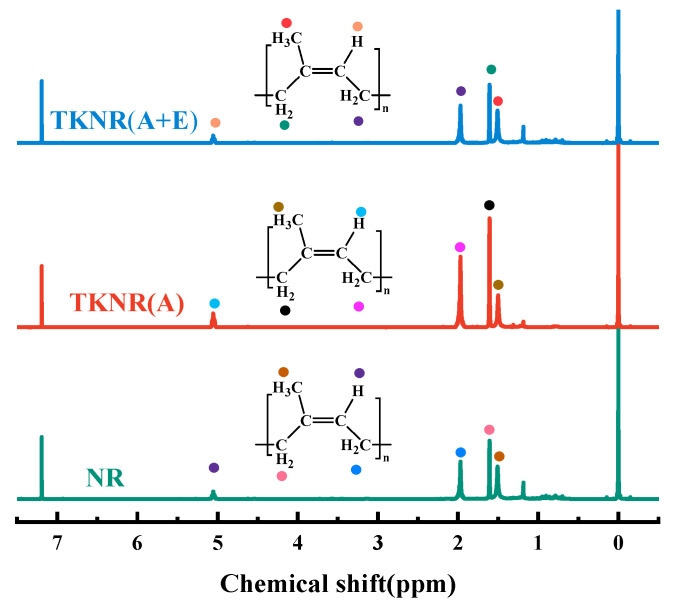
^1^H NMR spectra of NR and TKNR. (A + E) refers to the alkali–enzyme method for extracting TKNR, while (A) refers to the alkali method for extracting TKNR.

Figure 2 displays the ^1^H NMR spectra of NR and TKNR. The peaks at 0 ppm and 7.19 ppm correspond to the internal standard Tetramethylsilane (TMS, (CH_3_)_4_Si) and the deuterated chloroform solvent, respectively. For TKNR, the two−CH_2_ peaks appear at 1.61 ppm and 1.97 ppm, the C−H peak at 5.05 ppm, and the −CH_3_ peak at 1.50 ppm. These peak positions are nearly identical to those observed in NR, confirming that both materials exhibit the same cis-1,4-polyisoprene structure. However, the overall absorption intensities in TKNR are generally lower than in NR, suggesting potential partial degradation or reduced rubber hydrocarbon content as a result of the extraction process. Notably, the−CH_3_ and −CH_2_ peaks at 1.50 ppm and 1.61 ppm show significantly lower intensities in TKNR than in NR, reflecting the impact of extraction on molecular integrity, which leads to a reduction in characteristic group concentrations [26]. Increasing the duration and concentration of alkaline treatment reduces the molecular weight of TKNR and increases its polydispersity, thereby affecting the quality of TKNR.

### 2.2. Molecular Weight and Distribution Characteristics of Rubber

**Figure 3:**
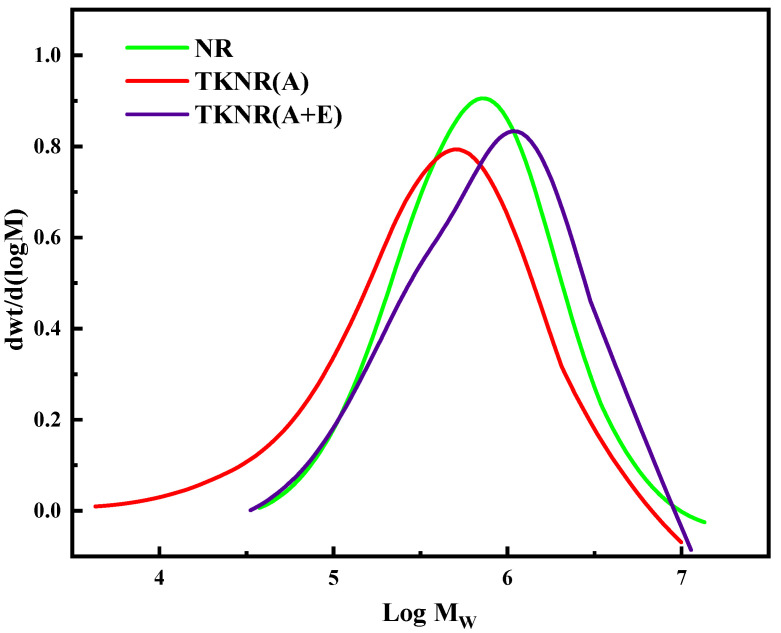
The molecular weight distribution of NR and TKNR.

Table 1 presents the molecular weights of NR and TKNR extracted by two methods, along with their molecular weight distributions shown in Figure 3. Comparisons of weight-average molecular weight (*Mw*), number-average molecular weight (*Mn*), and polydispersity index (PDI = *Mw/Mn*) reveal that NR exhibits an *M_w_* of 1.30 × 10^6^, Mn of 4.4 × 10^5^, and a PDI of 3.0, reflecting a broad and relatively uniform molecular weight distribution. In contrast, TKNR extracted by the alkali-based method (A) shows an *Mw* of 7.0 × 10^5^, *Mn* of 1.6 × 10^5^, and a PDI of 4.6, indicating a significantly lower molecular weight and a higher PDI. This suggests a broader and less uniform molecular weight distribution, likely due to an increase in low-molecular-weight components resulting from molecular chain degradation during extraction. On the other hand, TKNR extracted by the alkali-assisted enzymatic method (A + E) exhibits an *Mw* of 1.10 × 10^6^, *Mn* of 4.1 × 10^5^, and a PDI of 2.5, closer to the values observed for NR. This indicates that enzymatic extraction causes less molecular chain degradation, retains more high-molecular-weight components, and produces a more uniform molecular weight distribution [27].

The molecular weight distribution curves further corroborate these findings. The curve for TKNR extracted via the alkali-based method (A) shifts towards lower molecular weights and has a reduced peak, while the curve for TKNR extracted by the enzymatic method (A + E) closely resembles that of NR, with a higher peak and narrower distribution. This suggests that the enzymatic method better preserves the integrity of rubber molecular chains, leading to a more consistent molecular weight distribution. Thus, compared to the alkali-based method, the enzymatic method achieves superior retention of both molecular weight and distribution uniformity in TKNR.

### 2.3. Analysis of the Main Components of Rubber

**Figure 4:**
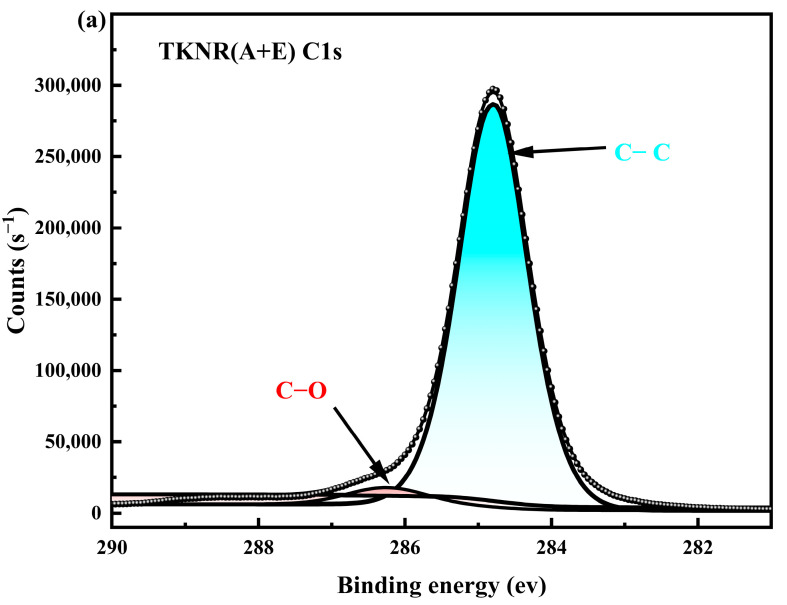

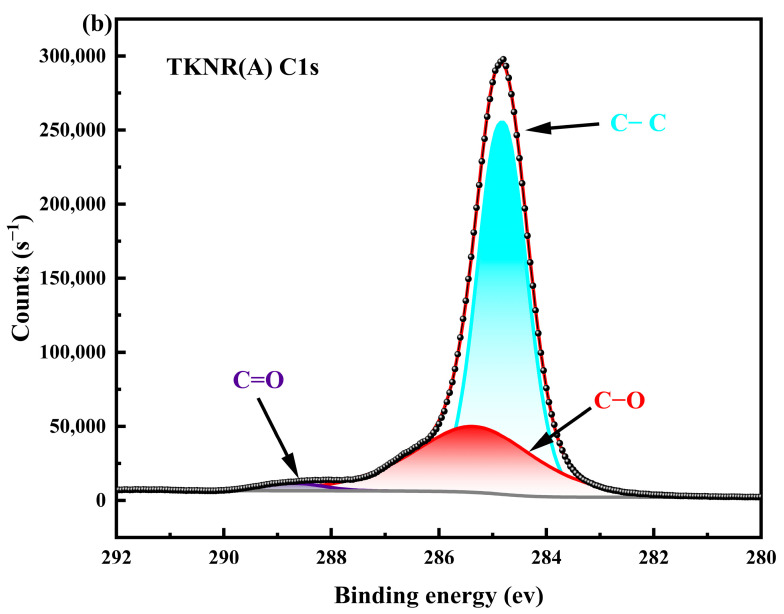
XPS C1s spectra: (**a**) TKNR(A + E); (**b**) TKNR(A).

Figure 4 presents the XPS C spectra, highlighting the influence of different extraction methods on the structure and chemical composition of TKNR. In Figure 4a, the C−C main peak at 285.0 eV for TKNR extracted via the alkaline–enzyme method aligns closely with that of NR, indicating effective preservation of the polyisoprene backbone structure. Additionally, the C−O secondary peak is relatively minor, and the C=O peak is nearly absent in the alkaline–enzyme sample, suggesting minimal formation of oxidation products during extraction. This indicates that the mild conditions of the alkaline–enzyme method effectively reduce molecular chain oxidation and degradation, preserving the chemical purity and structural integrity of TKNR [28].

Conversely, TKNR extracted using the alkaline method (Figure 4b) also retains the C−C main peak, but exhibits substantially larger C−O and C=O peak areas, indicating a higher degree of oxidation. This increase in oxidation likely results from the harsher extraction conditions of the alkaline method, leading to the formation of more oxidized compounds and impurities. Such oxidation can compromise the chemical purity of the material and potentially reduce its physical properties, such as elasticity and mechanical strength. Therefore, the alkaline–enzyme extraction method demonstrates clear advantages, significantly limiting oxidation-related side reactions and preserving the molecular integrity of TKNR, resulting in chemical characteristics more closely aligned with those of NR.

### 2.4. Analysis of Rubber Crosslink Density

**Figure 5:**
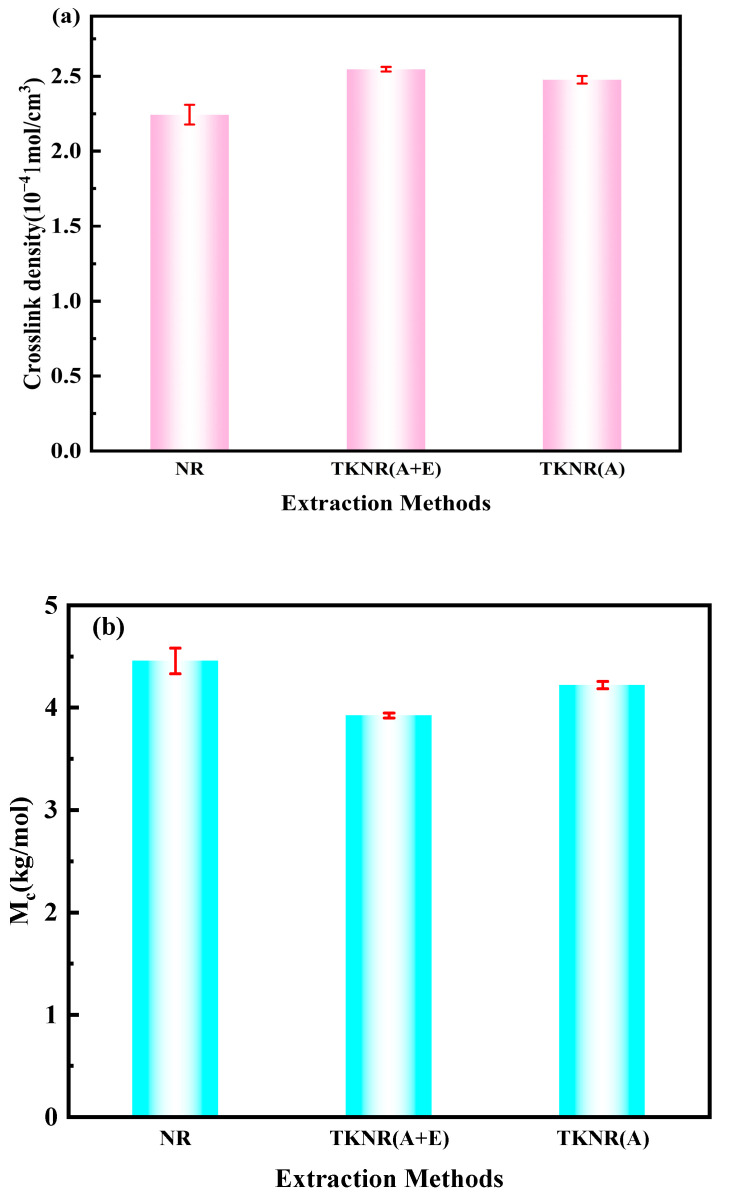
Crosslink networks of NR and TKNR: (**a**) crosslink density (v); (**b**) molecular weight between crosslinks (Mc).

Figure 5 illustrates the crosslink density and molecular weight between crosslinks (Mc) for NR and TKNR. Figure 5a,b depict the influence of various extraction methods on the crosslink network structures of TKNR and NR. The crosslink density (ν) of NR is measured at 2.24 × 10^−4^ mol/cm^3^, which is significantly lower than that of TKNR extracted via the alkaline–enzymatic method (2.55 × 10^−4^ mol/cm^3^) and the alkaline method (2.48 × 10^−4^ mol/cm^3^). This observation indicates that both extraction methods result in TKNR exhibiting a higher crosslink density compared to NR.

Moreover, the analysis of Mc reveals that NR has an Mc value of 4.46 kg/mol, which is greater than the values obtained for TKNR via the alkaline–enzymatic method (3.92 kg/mol) and the alkaline method (4.22 kg/mol). This finding suggests that NR possesses greater molecular chain spacing, reflecting a relatively looser crosslink network. In contrast, TKNR extracted using the alkaline–enzymatic method exhibits a smaller Mc and a higher ν, indicative of a denser crosslink network. These results demonstrate that TKNR extracted by both the alkaline–enzymatic and alkaline methods forms a tighter crosslink network than NR, with the alkaline–enzymatic method proving particularly effective in enhancing the crosslink density of TKNR.

### 2.5. Thermal Stability and Glass Transition Analysis of Rubber

**Figure 6:**
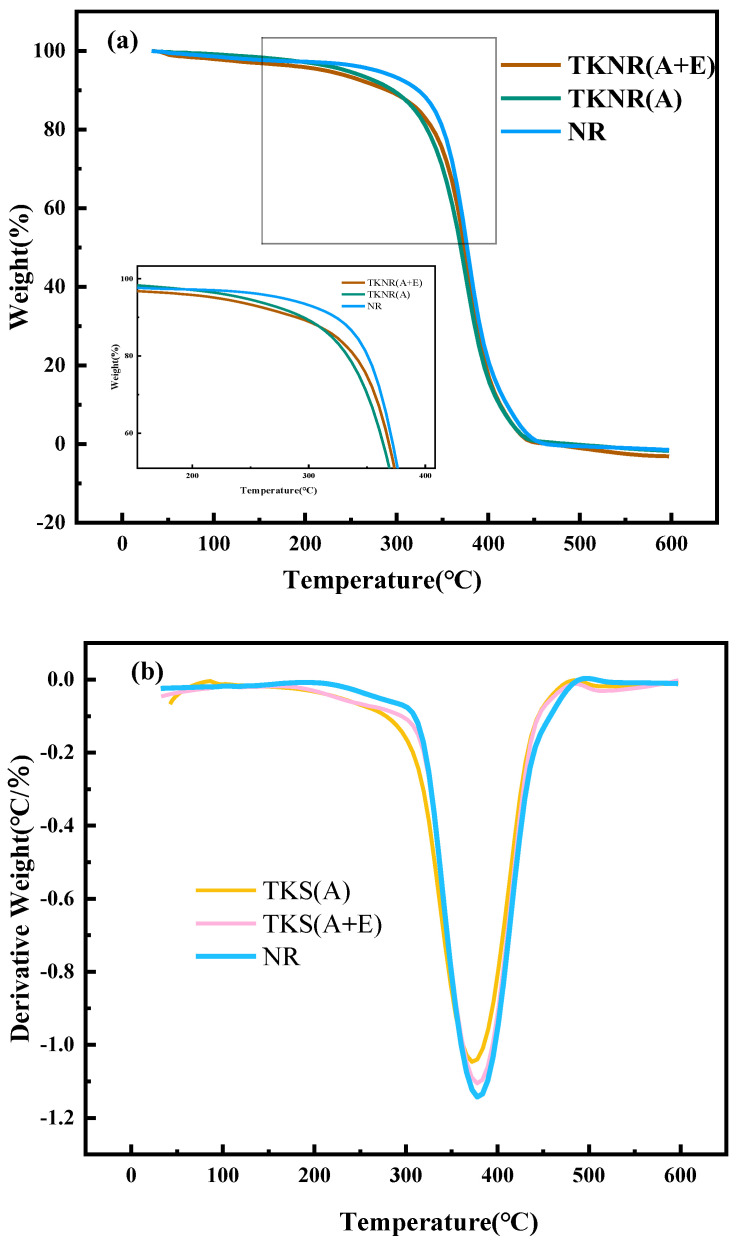
Thermal stability of NR and TKNR: (**a**) TG; (**b**) DTG.

Figure 6a,b present the thermogravimetric (TG) and derivative thermogravimetric (DTG) curves for NR, TKNR extracted via the alkaline method (A), and TKNR extracted via the alkaline–enzymatic method (A + E). The TG and DTG curves clearly indicate that the extraction method significantly influences the thermal stability of TKNR. NR exhibits a higher initial decomposition temperature, reflecting its superior thermal stability. In contrast, TKNR (A) and TKNR (A + E) exhibit lower initial decomposition temperatures. Table 2 demonstrates the thermo-oxidative degradation behavior of NR and TKNR samples, revealing that NR exhibits higher initial (T₀), peak (Tₚ), and final (T_f_) degradation temperatures compared to TKNR. Furthermore, the peak corresponding to the maximum weight loss rate in the DTG curve indicates that TKNR (A + E) experiences a slower thermal decomposition at elevated temperatures, further affirming its superior thermal stability. This improvement can be attributed to the crosslinking reactions induced by enzymatic treatment, which enhance the material’s thermal stability [29].

**Figure 7:**
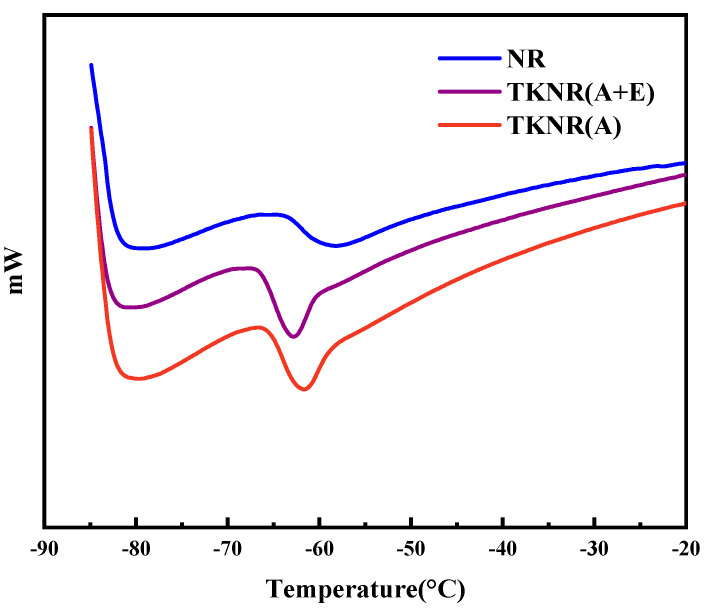
DSC curves of NR and TKNR.

Figure 7 illustrates the differential scanning calorimetry (DSC) curves for NR, TKNR (A), and TKNR (A + E). The DSC curves highlight the effects of various extraction methods on the glass transition temperature (Tg) and crystallinity of the samples. NR exhibited the highest glass transition temperature (Tg is −56.88 °C), indicating its relatively high molecular chain rigidity. In contrast, TKNR (A) (−60.94 °C) and TKNR (A + E) (−62.29 °C) demonstrated lower Tg values, with TKNR (A + E) showing the lowest Tg. This result suggests that enzymatic treatment reduces the rigidity of molecular chains and enhances their toughness.

Additionally, the enthalpy values of TKNR (A) and TKNR (A + E) are higher than that of NR, with the enthalpy (ΔH) of NR being 3.854 J/g, TKNR (A) 5.414 J/g, and TKNR (A + E) 6.155 J/g. TKNR (A + E) exhibits the highest enthalpy, corresponding to the highest degree of crystallinity (degree of crystallinity = experimentally measured enthalpy/theoretical melting enthalpy assuming 100% crystallinity, in units of J/g). This observation suggests that enzymatic treatment promotes a more orderly arrangement of molecular chains, thereby enhancing the material’s crystallinity [30]. Consequently, the alkaline–enzymatic extraction method not only effectively improves the thermal stability of TKNR but also enhances its overall thermal performance by increasing crystallinity.

## 3. Materials and Methods

### 3.1. Materials

Dried *TKS* roots were obtained from the Agricultural Science Research Institute of the Ili Autonomous Prefecture, Xinjiang (Urumqi, Xinjiang, China). Natural Rubber (NR) was sourced from the Jinlian Processing Branch of Hainan Natural Rubber Industry Group Co., Ltd. (Danzhou, Hainan, China). Potassium hydroxide (KOH Xinjiang 90%), dichloromethane (CH_2_Cl_2_, 98%), and tetrahydrofuran (THF, AR, ≥99.5%) were supplied by Guangzhou Chemical Reagent Factory (Guangzhou, Guangdong, China). Pectinase (500 U/mg), cellulase (400 U/mg), and sodium citrate buffer (0.5 M, pH 6.0) were purchased from Aladdin Reagent Co., Ltd. (Shanghai, China). Deuterated chloroform (D, 99.8%) was acquired from Shanghai Macklin Biochemical Technology Co., Ltd. (Shanghai, China), and toluene (AR, ≥99.5%) was obtained from Xilong Chemical Co., Ltd. (Xiangtan, Guangdong, China).

### 3.2. Methods

#### 3.2.1. Alkaline Method (A) for Extracting *Taraxacum kok-saghyz* Rubber

TKS roots are composed of root bark, root flesh, and root core, each with distinct TKNR concentrations: the root bark contains the highest TKNR content, the root flesh has a lower amount, and the root core is nearly devoid of TKNR. To enhance extraction efficiency and purity, the root bark and root core were processed separately. TKNR was extracted from the root bark using an alkaline treatment, while the minimal TKNR in the root flesh was recovered through acid treatment. First, 300 g of cleaned and pre-dried TKS roots (dried at 50 °C for 1–3 days) were boiled in water for 2 h at a solid/liquid ratio of 1:5, with this boiling process repeated three times. Following boiling, the root bark, flesh, and core were separated. The root bark was then treated with 3% potassium hydroxide (KOH) at 100 °C for 2 h, with intermittent stirring to promote reaction efficiency. The separated root flesh was treated with 3% H_2_SO_4_ at 100 °C for 3 h. Upon completion, the treated bark was thoroughly rinsed and subjected to centrifugation at 4000–5000 rpm for 15 min using a GL-21M centrifuge (Xiangyi Instrument Co., Ltd., Xiangtan, Hunan, China). The upper layer containing the floating rubber was collected and subsequently dried.

#### 3.2.2. Alkaline Treatment Combined with Enzymatic Hydrolysis (A + E) for Extracting *Taraxacum kok-saghyz* Rubber

To extract TKNR, 300 g of cleaned and dried *Taraxacum kok-saghyz* (TKS) roots were crushed and boiled in water at 100 °C for 30 min. The mixture was filtered through a 178 μm (80 mesh) sieve, and this process was repeated three times.

The filtered residue was dried at 50 °C for 24 h, and the collected filtrate, rich in inulin, was recovered by spray drying at 150 °C until completely dried. The dried residue was weighed, and KOH was added at a ratio of 60 mg KOH per gram of dry root, dissolved in 500 mL of deionized water. This mixture was treated at 120 °C for 30 min, then filtered (80 mesh sieve), and lignin was precipitated by acidifying the filtrate. The residue was subsequently washed with 5 L of deionized water, followed by an additional 2 L, and allowed to stand at 4 °C overnight to remove residual alkali ions. After further filtration and washing, the residue was suspended in 1.5 L of deionized water, and pectinase and cellulase were added at a 1.5:1 ratio (42 mg pectinase (500 U/mg) and 27.5 mg cellulase (400 U/mg) per gram of dry root). The pH of the mixture was adjusted using 0.5 M sodium citrate buffer (pH 5.5). The mixture was then sealed in a beaker and stirred magnetically at 50 °C and 200 rpm for 48 h.

**Figure 8:**
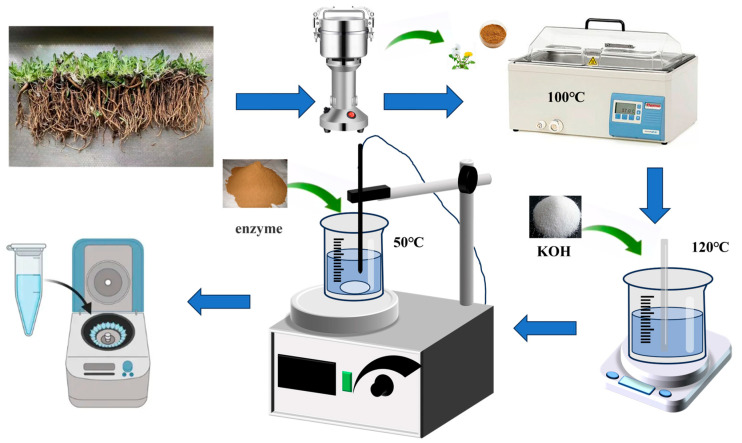
Flowchart of TKNR extraction using the combined alkaline treatment and enzymatic hydrolysis method.

Upon completion of the enzymatic reaction, the mixture was centrifuged at 5000 rpm for 30 min at 4 °C. The upper layer containing the floating rubber was carefully collected, dried, and stored. An illustration of the extraction process is presented in Figure 8.

### 3.3. Characterizations

#### 3.3.1. Fourier Transform Infrared Spectroscopy (FTIR)

FTIR analysis was performed using a PerkinElmer Spectrum One FTIR spectrometer (Waltham, MA, USA). A sample of 5–10 mg was dissolved in dichloromethane, and the resulting solution was applied to a potassium bromide (KBr) pellet. The solvent was evaporated under an infrared lamp, after which the KBr pellet was positioned in the transmission accessory. The analysis was conducted in transmission mode (TR) over a scan range of 4000–500 cm^−1^, with a total of 32 scans recorded.

#### 3.3.2. Nuclear Magnetic Resonance Spectroscopy (^1^H NMR)

^1^H NMR analysis was conducted using a Bruker AVANCE NEO 400 MHz spectrometer (Bruker BioSpin AG, Basel, Switzerland). A sample of 5–10 mg was dissolved in deuterated chloroform (CDCl_3_), filtered through a 0.22 μm membrane, and subsequently transferred into an NMR tube. The analysis was performed to obtain the ^1^H NMR spectrum.

#### 3.3.3. Gel Permeation Chromatography (GPC)

Molecular weight and molecular weight distribution were analyzed using a Waters 1515 GPC system (Waters, Milford, MA, USA). A sample of 5–10 mg was dissolved in tetrahydrofuran (THF) and protected from light in an amber bottle. After complete dissolution, the solution was filtered through a 0.22 μm membrane and analyzed for 45 min to determine molecular weight and polydispersity. The mobile phase is tetrahydrofuran, polystyrene is used as the standard sample, the flow rate is 1 mL/min, and the molecular weight range of the chromatographic column is 1–2,000,000.

#### 3.3.4. X-Ray Photoelectron Spectroscopy (XPS)

XPS measurements were conducted using a Thermo Fisher Scientific K-Alpha XPS spectrometer (Waltham, MA, USA). A sample of 20–30 mg was cut into small pieces and adhered to the sample holder using conductive tape. C 1s spectra were recorded to analyze the composition of the rubber.

#### 3.3.5. Crosslink Density Analysis

Crosslink density was determined using a VTNMR20-010V-T crosslink density analyzer (Shanghai Niumag Corporation, Shanghai, China). A sample of appropriate size was placed into a glass tube and stabilized in the magnetic field before measurement. Crosslink density and rubber network structure were calculated using the XLD-2 model.

#### 3.3.6. Thermogravimetric Analysis (TGA)

TGA was performed using a Mettler Toledo TGA/DSC 1/1100 thermogravimetric analyzer (Zurich, Switzerland). The sample was heated from 25 °C to 600 °C at a rate of 10 °C/min under a nitrogen flow of 60.0 mL/min to assess thermal stability.

#### 3.3.7. Differential Scanning Calorimetry (DSC)

DSC analysis was conducted using a METTLER TOLEDO DSC822e differential scanning calorimeter (Zurich, Switzerland). The sample was heated from −85 °C to 100 °C at a rate of 10 °C/min under a nitrogen flow of 60.0 mL/min to determine the glass transition temperature and crystallinity.

## 4. Conclusions

The FTIR and ^1^H NMR analyses indicate that both the alkaline and alkaline–enzymatic extraction methods successfully preserved the cis-1,4-polyisoprene backbone structure of TKNR, which is similar to that of NR. However, TKNR extracted via the alkaline–enzymatic method exhibited less degradation of molecular chains, with smaller losses in the intensity of C−H deformation and methyl symmetric deformation vibrations, suggesting better protection of the molecular chain integrity. Additionally, XPS results revealed that the alkaline–enzymatic method effectively minimized oxidation side reactions that typically occur under harsh extraction conditions, producing fewer oxidation products, and the chemical properties of the extracted TKNR were found to be closer to those of NR. Molecular weight and distribution analysis further demonstrated that the alkaline–enzymatic extracted TKNR exhibited a *Mw* and PDI closer to NR, with lower molecular chain degradation. Gel content analysis showed that the alkaline–enzymatic method provided greater stability in maintaining the rubber network structure. Crosslink density and molecular weight between crosslinks analyses indicated that the crosslink network was denser and more uniform compared to the alkaline method. Furthermore, thermogravimetric analysis and differential scanning calorimetry confirmed that TKNR obtained via this method had enhanced thermal stability and a lower glass transition temperature. Overall, the alkaline–enzymatic extraction method not only achieves a chemical purity close to that of NR, but also offers superior molecular structure integrity and physical properties compared to the alkaline method, making it the most promising approach for extracting TKNR to date.

## Figures and Tables

**Table 1:** Molecular weights and polydispersity index (PDI) of NR and TKNR.

Extraction Methods	*M*w	*M*n	PDI
NR	13.0 × 10^5^	4.4 × 10^5^	3.0
TKNR(A)	7.0 × 10^5^	1.6 × 10^5^	4.6
TKNR(A + E)	11.0 × 10^5^	4.1 × 10^5^	2.5

**Table 2:** Thermo-oxidative degradation characteristic temperatures of NR and TKNR.

Samples	T_0_ (°C)	T_p_ (°C)	T_f_ (°C)
NR	342.58	377.92	464.28
TKNR(A)	319.47	372.20	452.73
TKNR(A+E)	307.44	377.68	446.97

## Data Availability

The original contributions presented in this study are included in the article. Further inquiries can be directed to the corresponding authors.

## References

[1] Yamashita S., Takahashi S. (2020). Molecular Mechanisms of Natural Rubber Biosynthesis. Annu. Rev. Biochem..

[2] Demirbas M.F., Balat M. (2006). Recent advances on the production and utilization trends of bio-fuels: A global perspective. Energy Convers. Manag..

[3] Yang N., Yang D., Yu X., Xu C. (2023). Multi-omics-driven development of alternative crops for natural rubber production. J. Integr. Agric..

[4] Iaffaldano B., Cardina J., Cornish K. (2018). Hybridization Potential between the Rubber Dandelion and Common Dandelion. Ecosphere.

[5] Gabit B., Karina G., Gulnar G., Kairat U., Yaroslav G., Aleksey C., Bulat K. (2022). Multilocus DNA Polymorphism of Some Rubber-bearing Dandelions (*Taraxacum* spp.) of Russia and Kazakhstan. Genet. Resour. Crop Evol..

[6] Keener H.M., Shah A., Klingman M., Wolfe S., Pote D., Fioritto R. (2018). Progress in Direct Seeding of an Alternative Natural Rubber Plant, *Taraxacum kok-saghyz* (L.E. Rodin). Agronomy.

[7] Saeedi F., Naghavi M.R., Sabokdast M., Jariani P. (2023). *Taraxacum kok-saghyz* L.E. Rodin as a Novel Potential Source of Natural Rubber in Iran: A Good Candidate for Commercial Use. Iran. Polym. J..

[8] Ramirez-Cadavid D.A., Cornish K., Michel F.C. (2017). *Taraxacum kok-saghyz* (TK): Compositional Analysis of a Feedstock for Natural Rubber and Other Bioproducts. Ind. Crops Prod..

[9] Abdul Ghaffar M.A., Meulia T., Cornish K. (2016). Laticifer and Rubber Particle Ontogeny in *Taraxacum kok-saghyz* (Rubber Dandelion) Roots. Microsc. Microanal..

[10] King-Smith N., Molnar K., Blakeslee J.J., McMahan C.M., Pillai A.S., Mutalkhanov M., Puskas J.E., Cornish K. (2023). Extractable Latex Yield from *Taraxacum kok-saghyz* Roots is Enhanced by Increasing Rubber Particle Buoyancy. Ind. Crops Prod..

[11] Yuan B.X., Ding G.H., Ma J.J., Wang L.L., Yu L., Ruan X.Y., Zhang X.Y., Zhang W.F., Wang X.C., Xie Q.L. (2020). Comparison of Morphological Characteristics and Determination of Different Patterns for Rubber Particles in Dandelion and Different Rubber Grass Varieties. Plants.

[12] Gao S.K., Guo M.M., Gao J.Q., Huang Z.J., Gan M., Zhang J.C., Dong Y.Y. (2024). Rapid Determination of Rubber Content Using a Pyrolyzer Hyphenated with a Miniaturized Mass Spectrometer. Separations.

[13] Martirosyan L.Y., Goldberg V.M., Barashkova I.I., Kasparov V.V., Martirosyan Y.T., Motyakin M.V., Gaydamaka S.N., Varfolomeev S.D. (2023). Quantitative Determination of Natural Rubber Content of *Taraxacum kok-saghyz* E. Rodin Plants Using Spin Probe Method of Electron Paramagnetic Resonance Spectroscopy. Biophysics.

[14] Ramirez-Cadavid D.A., Valles-Ramirez S., Cornish K., Michel F.C. (2018). Simultaneous Quantification of Rubber, Inulin, and Resins in *Taraxacum kok-saghyz* (TK) Roots by Sequential Solvent Extraction. Ind. Crops Prod..

[15] Ramirez-Cadavid D.A., Cornish K., Hathwaik U., Kozak R., McMahan C., Michel F.C. (2019). Development of Novel Processes for the Aqueous Extraction of Natural Rubber from *Taraxacum kok-saghyz* (TK). J. Chem. Technol. Biotechnol..

[16] Ramirez-Cadavid D.A., Hathwaik U., Cornish K., McMahan C., Michel F.C. (2022). Alkaline Pretreatment of *Taraxacum kok-saghyz* (TK) Roots for the Extraction of Natural Rubber (NR). Biochem. Eng. J..

[17] Sikandar S., Ujor V.C., Ezeji T.C., Rossington J.L., Michel F.C., McMahan C.M., Ali N., Cornish K. (2017). STm: A Source of Thermostable Hydrolytic Enzymes for Novel Application in Extraction of High-quality Natural Rubber from *Taraxacum kok-saghyz* (Rubber Dandelion). Ind. Crops Prod..

[18] Zhao S., Jie X., Ma Z., Wang Z., Zhang J.C., Li Y.S., Nie Q.H., Ma Y. (2023). Preparation of Rubber and Biofuel Ethanol Simultaneously by the Yeast Fermentation Process. ACS Omega.

[19] Xiao R., Shen Z.L., Si R.Z., Polaczyk P., Li Y.C., Zhou H.Y., Huang B.S. (2022). Alkali-activated Slag (AAS) and OPC-based Composites Containing Crumb Rubber Aggregate: Physico-mechanical Properties, Durability and Oxidation of Rubber upon NaOH Treatment. J. Clean. Prod..

[20] Zhuo Y., Zhang C., Zhao Y., Hu B., Liao S., Liao X.-X. (2022). Composition Properties of Rubber from Parts of *Taraxacum kok-saghyz* Roots. J. Rubber Res..

[21] Piccolella S., Sirignano C., Pacifico S., Fantini E., Daddiego L., Facella P., Lopez L., Scafati O.T., Panara F., Rigano D. (2023). Beyond Natural Rubber: *Taraxacum kok-saghyz* and *Taraxacum brevicorniculatum* as Sources of Bioactive Compounds. Ind. Crops Prod..

[22] Buranov A.U., Elmuradov B.J. (2010). Extraction and Characterization of Latex and Natural Rubber from Rubber-bearing Plants. J. Agric. Food Chem..

[23] Chen X., Ji H., Zhang C., Liu A. (2019). Optimization of Extraction Process from *Taraxacum officinale* Polysaccharide and Its Purification, Structural Characterization, Antioxidant and Anti-tumor Activity. J. Food Meas. Charact..

[24] Olsen K.M., Li L.-F. (2018). Rooting for New Sources of Natural Rubber. Natl. Sci. Rev..

[25] Liu S.Q., Chen Y.H., Han D.R., Tian X.F., Ma D.L., Jie X., Zhang J.C. (2024). Extraction Process and Characterization of *Taraxacum kok-saghyz* (TKS) Latex. Heliyon.

[26] Salehi M., Bahmankar M., Naghavi M.R., Cornish K. (2022). Rubber and Latex Extraction Processes for *Taraxacum kok-saghyz*. Ind. Crops Prod..

[27] Nun-anan P., Wisunthorn S., Pichaiyut S., Vennemann N., Kummerlöwe C., Nakason C. (2020). Influence of alkaline treatment and acetone extraction of natural rubber matrix on properties of carbon black filled natural rubber vulcanizates. Polym. Test..

[28] Schmidt T., Lenders M., Hillebrand A., van Deenen N., Munt O., Reichelt R., Eisenreich W., Fischer R., Prüfer D., Gronover C.S. (2010). Characterization of Rubber Particles and Rubber Chain Elongation in *Taraxacum kok-saghyz*. BMC Biochem..

[29] Ikeda Y., Junkong P., Ohashi T., Phakkeeree T., Sakaki Y., Tohsan A., Kohjiya S., Cornish K. (2016). Strain-induced Crystallization Behaviour of Natural Rubbers from Guayule and Rubber Dandelion Revealed by Simultaneous Time-resolved WAXD/tensile Measurements: Indispensable Function for Sustainable Resources. RSC Adv..

[30] Musto S., Barbera V., Maggio M., Mauro M., Guerra G., Galimberti M. (2016). Crystallinity and Crystalline Phase Orientation of Poly(1,4-cis-isoprene) from *Hevea brasiliensis* and *Taraxacum kok-saghyz*. Polym. Adv. Technol..

